# An X-Band Class-J GaN MMIC Power Amplifier with Well-Designed In-Band Output Power Flatness

**DOI:** 10.3390/mi16010087

**Published:** 2025-01-13

**Authors:** Bangjie Zheng, Zhiqun Cheng, Zhiwei Zhang, Ruizhe Zhang, Tingwei Gong, Chao Le

**Affiliations:** 1 School of Electronics and Information, Hangzhou Dianzi University, Hangzhou 310018, China; 2 School of Information Engineering, Xinjiang Institute of Technology, Aksu 843100, China; 3 State Key Laboratory of Millimeter-Waves, Nanjing 210096, China

**Keywords:** power amplifier (PA), MMIC, GaN, X-band, Class-J

## Abstract

This paper presents an X-band high-power GaN MMIC power amplifier (PA). To balance efficiency, output power, and saturated power flatness, the load-line theory is employed to analyze and validate the power variation trends within an extended continuous Class B/J (CCBJ) impedance space. Theoretical constant power contours are plotted within this space. An L-C impedance matching network is used to match the amplifier’s output impedance to the overlapping region of the 0.5 dB constant power contour and the CCBJ impedance space, significantly improving the in-band power flatness of the PA based on the CCBJ design approach. Additionally, an RC parallel structure is integrated into the interstage matching network to maximize gain while ensuring stability. The proposed PA, implemented using a 0.25 µm commercial GaN process, achieves a saturated output power of 47–47.6 dBm with in-band fluctuations within ± 0.3 dB, a power gain of 27.0–27.8 dB, and an efficiency of 40–45.5% across the X-band.

## 1. Introduction

The X-band (8–12 GHz) power amplifier (PA) is crucial for applications in radar systems, phased array technologies, satellites, and communication systems [1]. With the increasing demand for higher performance in terms of saturated output power (*P*_sat_), gain, efficiency, and size, power amplifiers have become a key limiting factor in the overall system performance. Among the various technologies available, gallium nitride (GaN) MMICs have emerged as the preferred choice for high-performance PAs due to their superior breakdown voltage, high thermal conductivity, and excellent power density [2]. Over the past decade, significant progress has been made in GaN MMIC PAs operating across various modes and frequency bands [3,4,5]. These advancements have highlighted the potential of GaN-based designs to push the boundaries of power, efficiency, and bandwidth for high-frequency applications.

The development of various operational modes for power amplifiers has significantly contributed to improving the performance of these devices. For example, Class-F amplifiers, which use a combination of harmonic termination techniques, are well known for their high efficiency due to the elimination of power losses in harmonics [6]. Similarly, Class-E amplifiers, with their resonant load network, achieve high efficiency by operating with zero voltage and current overlap, thus minimizing the losses associated with switching transitions [7]. More recently, the Class-GF mode has been introduced, which combines the advantages of Class-F and Class-E amplifiers while further extending the bandwidth and enhancing the efficiency by optimizing both fundamental and harmonic components [8]. These techniques have been successful in achieving high power output and efficiency, but they often face limitations in maintaining a broad impedance space, particularly when aiming for multi-octave bandwidths.

The Class-J mode, introduced by Cripps in 2009 [9], was a significant breakthrough as it extended Class-B into a continuous mode, offering a broader impedance space for both fundamental and second harmonic frequencies. This feature provides more flexibility for power amplifier designs, enabling better performance in terms of efficiency and output power across a wider range of frequencies. However, while Class-J offered several advantages, the need to optimize both the fundamental and harmonic impedance spaces for high-performance designs remained a challenge. In response to this, the resistive–reactive Class-J (RRCJ) mode was developed [10,11], incorporating a resistive factor (*β*) into the voltage waveform. This modification extended the impedance space for the fundamental frequency to the left and for the second harmonic to the right on the Smith chart, creating an overlap that significantly improved efficiency and output power across broader frequency bands.

Further advancing this approach, the extended resistive continuous class B/J (CCBJ) mode [12] introduced an additional factor (*γ*) that further refined the impedance space, allowing designers to achieve even higher efficiency while minimizing distortion. This mode proved especially effective in extending the bandwidth and improving the performance of GaN-based power amplifiers. However, according to load-pull results, a significant reduction in saturated power is observed within the expanded continuous Class-B/J impedance space. This behavior deviates from the ideal conditions predicted by theory. This discrepancy arises due to the fact the voltage swing limitation in actual PAs is neglected during the manipulation of the voltage waveform. As a result, despite these advancements, achieving a well-balanced design that ensures high power flatness, stable gain, and efficiency across the entire X-band remains a challenging task, especially when dealing with the complex impedance-matching requirements of modern communication and radar systems.

In this context, the work presented in this paper leverages the benefits of the CCBJ mode while addressing its limitations for achieving good power flatness. Firstly, based on load-line theory, the impedance space of equal power contours is calculated and analyzed, and for the first time, the output power variation trend within the CCBJ theory impedance space is explored. Secondly, by combining the CCBJ impedance space with the calculated constant power contours, the target impedance space for designing high-efficiency PAs with minimum in-band power fluctuation is derived. Finally, a GaN MMIC power amplifier operating in the 8–12 GHz range is designed and fabricated. The proposed amplifier achieves high saturated power and efficiency and demonstrates significant advantages in in-band power flatness when compared to similar amplifiers reported in the literature.

## 2. Analysis of the Proposed Design Strategy

The voltage waveform of Class-J mode [9] is defined by Equation (1):

(1) $$V_{J}(\theta )=(1-cos\theta )\cdot (1-\alpha \;sin\theta )$$

where *α* (−1 ≤ *α* ≤ 1) represents the continuous mode parameter and *θ* represents the phase angle of the output voltage related to frequency. As long as the value of *α* is within the range of [−1,1], the efficiency remains at the ideal Class-J efficiency of 78.7%, while the *P*_sat_ remains unchanged.

The voltage waveform of the RRCJ mode [12] adds a resistive factor $\left(1+\beta \;cos\theta \right)$ to Equation (1), forming Equation (2).

(2) $$V_{RRCJ}(\theta )=(1-cos\theta )\cdot (1-\alpha \;sin\theta )\cdot (1-\beta \;cos\theta )$$

Here, *β* (0 ≤ *β* ≤ 0.38) represents the resistive modulation coefficient. The addition of the resistive factor extends the fundamental impedance of Class-J to the left from the center, while the second harmonic impedance is extended to the right from the edge of the Smith chart, thereby merging the fundamental and second harmonic impedance spaces.

The voltage waveform of the CCBJ mode is given by Equation (3):

(3) $$V_{CCBJ}(\theta )=(1-cos\theta )\cdot (\gamma -\alpha \;sin\theta )\cdot (1-\beta \;cos\theta )$$

which adds the *γ* factor (1≤γ≤1/(1−β)) to the RRCJ mode, extending the fundamental impedance space from the center to the right. The value of *γ* controls the extent of the impedance space expansion. The drain-source current (*i*_ds_) for the three modes mentioned above is defined as Equation (4).

(4) $$i_{ds}\left(\theta \right)=1+\sum_{k=1}^{\infty }i_{k}cos\;k\theta =1+\frac{\pi }{2}cos\theta +\frac{2}{3}cos2\theta \ldots$$

According to formulae (3) and (4) in Ref. [12], the fundamental and second harmonic impedances of the CCBJ mode are derived as shown in Equation (5).

(5) $$Z_{1}=R_{opt}\left[\gamma \left(1-\beta \right)+j\alpha (1-0.25\;\beta )\right],\;\;\;Z_{2}=\frac{3\pi R_{opt}}{8}\left[\gamma \beta +j\alpha (1-\beta )\right]$$

**Figure 1:**
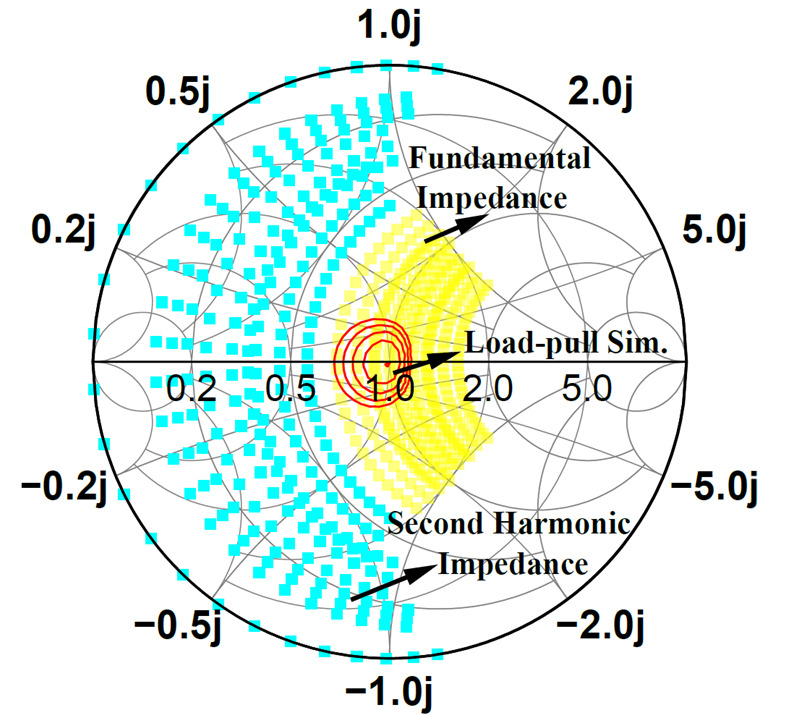
The impedance space of CCBJ and the load-pull simulation results.

The corresponding impedance space is illustrated in Figure 1, with parameter ranges defined as −1≤α≤1,0≤β≤0.38,1≤γ≤1.6. The yellow area represents the fundamental impedance, defined as *Z*_1_, and the blue area represents the second harmonic impedance, defined as *Z*_2_.

Although efficiency within the impedance space has been extensively studied, output power analysis remains limited. As shown in Figure 1, the red circles represent power contours from load-pull simulations at 12 GHz using the GaN transistor employed in this work (normalized to *R*_opt_), with power steps set to 0.3 dB and the harmonic impedance configured as open. Within the CCBJ-defined impedance space, saturated power fluctuations exceed ± 1.2 dB. This variation causes significant power fluctuations across the amplifier’s bandwidth.

To address this, an analysis based on the classic load-line theory is conducted. The concept of optimal power impedance (*R*_opt_) was first introduced in the load-line theory proposed in Ref. [13] and has since been widely applied in various power amplifier theories. During PA operation, the output voltage is limited by the drain supply voltage (*V*_dc_), while the maximum drain-source current (*I*_max_) is constrained by the product of transistor size and its power density. When either the voltage or current exceeds these physical limits, power compression occurs. Assuming that the output voltage (*V*_DS_) and current (*I*_D_) expressions are given as in Equation (6):

(6) $$V_{DS}=V_{dc}\left(1-\sin\theta \right),I_{D}=\frac{I_{max}}{2}\left(\sin\theta +1\right),\;{\;I}_{dc}=\frac{I_{max}}{2}$$

a load impedance, *R*_opt,_ exists that allows both the voltage and current amplitudes to reach their maximum values, thereby delivering the maximum power *P*_opt_, as shown in Equation (7).

(7) $$R_{opt}=\frac{V_{dc}}{I_{max}/2}=\frac{V_{dc}}{I_{dc}},P_{opt}=\frac{1}{2}\cdot \frac{V_{dc}}{I_{dc}}$$

When the load impedance decreases to $R_{opt}/p\;(p\geq 1)$, defined as *R*_LO_, the voltage swing must reduce to prevent the current swing from exceeding *I*_max_. Conversely, when the load impedance increases to ${pR}_{opt}$, defined as *R*_HI_, the current swing must decrease to ensure that the voltage swing remains within 2*V*_dc_. The corresponding output powers, *P*_LO_ and *P*_HI_, are given in Equation (8).

(8) $$R_{LO}=\frac{R_{opt}}{p},\;R_{HI}=p\cdot R_{opt}\;,\;{\;\;P}_{LO}=P_{HI}=\frac{P_{opt}}{p}$$

**Figure 2:**
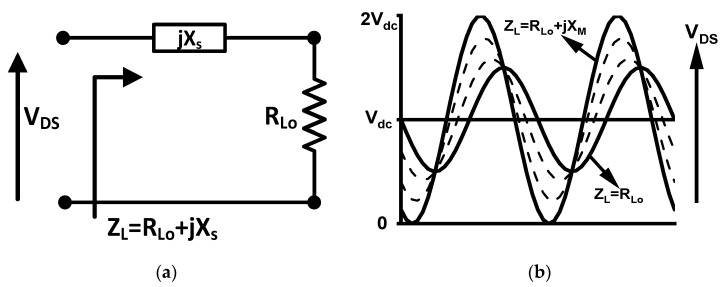
(**a**) The impedance of the load, *Z*_L_; (**b**) variations of *V*_ds_ with *Z*_L_.

To analyze the impact of the reactance on power, the series reactance *jX_s_* is added to the load resistance *R*_LO_, as shown in Figure 2a. As *X_s_* increases, the phase and amplitude of the voltage waveform *V*_ds_ change, as illustrated in Figure 2b. Notably, when *X*_s_ reaches a certain level, denoted as *X*_m_, the voltage amplitude returns to its physical limit of 2*V*_dc_. However, if *X*_s_ continues to increase, the voltage amplitude will exceed this limit, leading to power compression. From these calculations, the conclusions presented in Equation (9) can be derived:

(9) $$P_{RF}=\frac{P_{opt}}{p}\;\left(-X_{m}\leq X_{s}\leq X_{m}\right),\sqrt{{R_{LO}}^{2}+{X_{m}}^{2}}=R_{opt}$$

which shows that when the real part of the impedance is set to *R*_LO_ and the imaginary part varies between −*X*_m_ and *X*_m_, the output power (*P*_RF_) remains constant. Similarly, when the load resistance equals *R*_HI_, adding a parallel reactance results in an analogous conclusion; the detailed derivation process can be found in Ref. [13].

**Figure 3:**
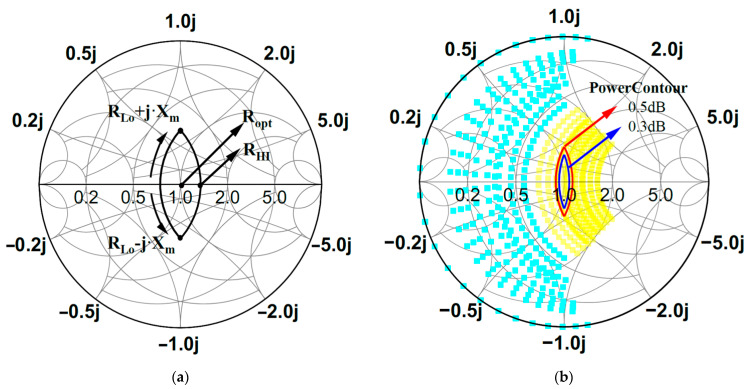
(**a**) Consistent power circle of the load-line theory; (**b**) 0.3 dB and 0.5 dB power contours within the impedance space of CCBJ.

Based on the above analysis, by plotting the impedance space on the Smith chart, a closed power contour curve can be obtained, as shown in Figure 3a. By combining the load-line power contours with the theoretical impedance space of CCBJ, a more realistic representation of power variations within the CCBJ-mode impedance space can be obtained. As shown in Figure 3b, by plotting the CCBJ-mode impedance space alongside the 0.3 dB and 0.5 dB power contours on the same Smith chart, the impedance regions where the power fluctuations are less than 0.3 dB and 0.5 dB within the CCBJ-type impedance space can be identified. As a result, designing a power amplifier within the overlapping impedance space leads to a highly efficient class-J power amplifier with excellent in-band saturation power flatness.

## 3. Circuit Design and Implementation

**Figure 4:**
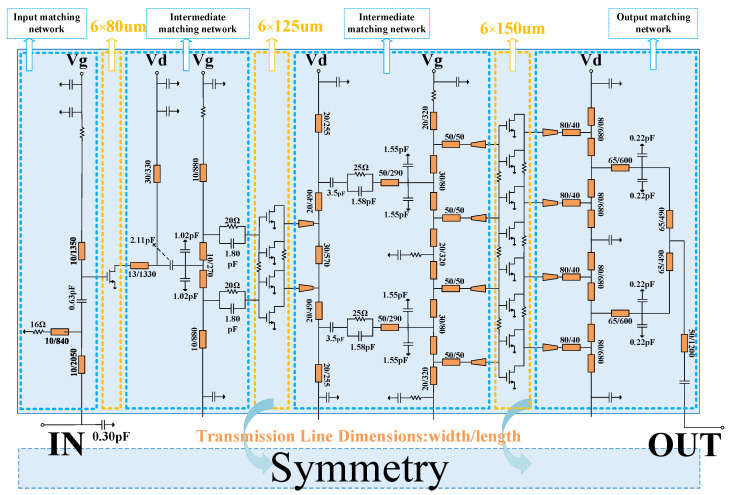
Schematic of the designed PA.

**Figure 5:**
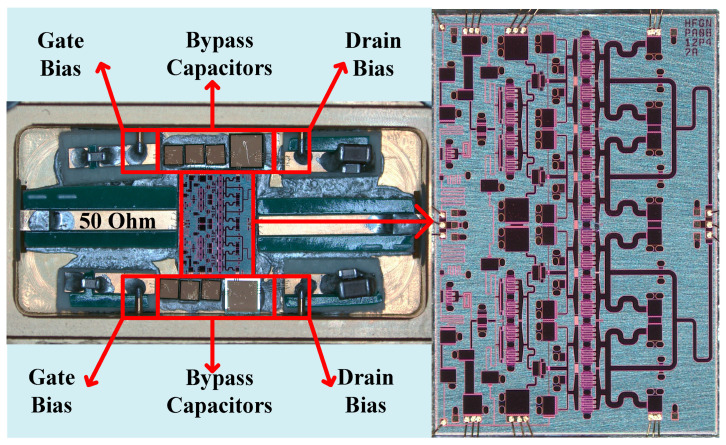
Photograph of the fabricated PA.

For validation, an X-band Class-J MMIC PA is designed using a 0.25 µm commercial GaN HEMT process. The schematic of the fabricated PA is shown in Figure 4 and its corresponding photograph is presented in Figure 5. The drain DC bias voltage (*V*_d_) of all three stages is 28 V, while the gate DC bias voltage (*V*_g_) is set to −2.2 V. The transistor sizes for the three stages are 2 × 6 × 80 µm, 8 × 6 × 80 µm, and 16 × 6 × 150 µm, respectively. To ensure stability, a balancing resistor is added between every two pairs of transistors in the second and third stages, and resistors are also placed on the gates of all transistors, as shown in Figure 4. The layout adopts a fully symmetrical structure, with a top-and-bottom symmetric power supply configuration. All capacitors without labeled values in Figure 4 are bypass capacitors. As shown in the blue box in Figure 4, the matching circuit is divided into four parts: the output matching network, two intermediate matching networks, and the input matching network. The details of each part are described below.

### 3.1. Output Matching Network

Based on the analysis in Section 2, the output fundamental impedance needs to be matched to the overlapping region of the equal power contour and the CCBJ-type impedance space. Through our calculations, the real part of the fundamental load impedance is determined to have a range of [0.89, 1.12], while the imaginary part has a range of [−0.45, 0.45]. The corresponding ranges for *α*, *β*, and *γ* are [−0.4, 0.4], [0, 0.13], and [1, 1.1].

**Figure 6:**
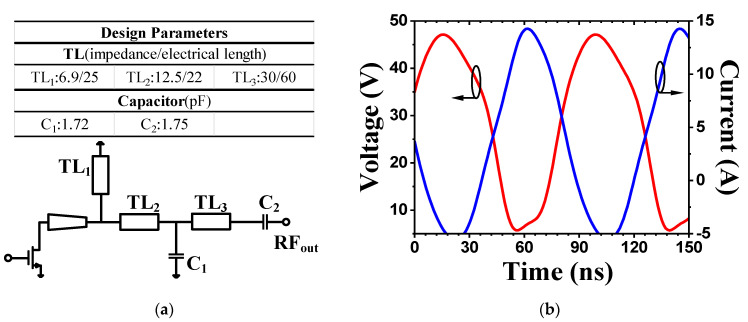
(**a**) Output harmonic tuning network; (**b**) simulated drain voltage and current waveforms.

**Figure 7:**
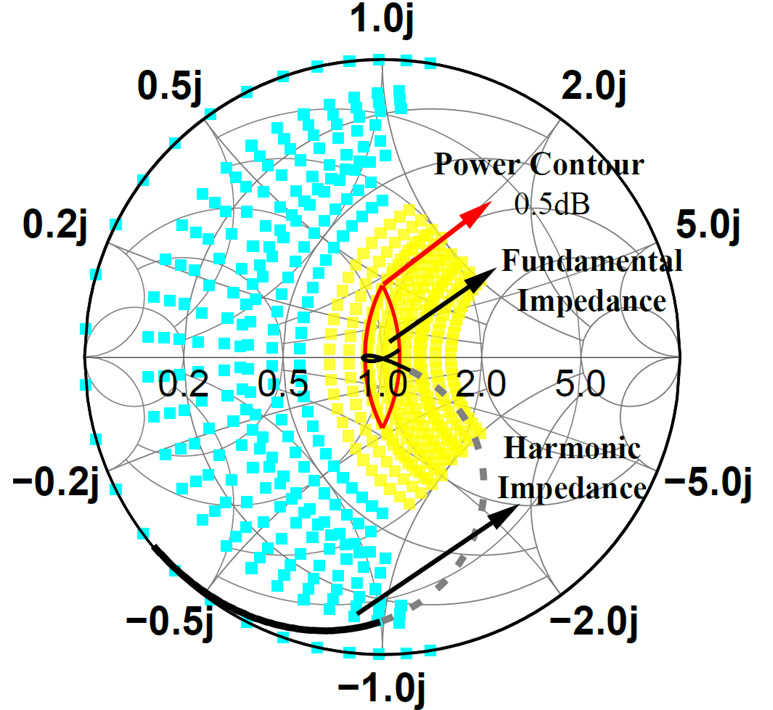
Simulated impedance of the output matching network.

To match the output impedance to the target impedance space shown in Figure 3b, an L-C-L three-section impedance matching network was employed. The equivalent lumped-element circuit is illustrated in Figure 6a, with *TL*_1_ serving as an RF chock connected to the drain voltage supply, while *C*_2_ serves as a DC-blocking capacitor. By combining calculations with ADS optimization, the parameters of the matching network can be determined, as shown in the table in Figure 6a. The simulated impedance trajectory of the final output matching network is represented by the black line in Figure 7. It is evident that the fundamental impedance falls perfectly within the target impedance space, while the harmonic impedance also lies within the CCBJ-defined impedance space. The simulation results show that the implemented impedance matching network achieves an insertion loss between 0.4 dB and 0.48 dB across the operating frequency band. The large-signal simulation results further demonstrate that the output stage delivers power in the range of 47.8 dBm to 48.2 dBm, exhibiting excellent in-band flatness that is consistent with the theoretical analysis. The simulated drain voltage and current waveforms of the output stage are shown in Figure 6b, aligning well with the theoretical waveforms of the CCBJ mode.

### 3.2. Intermediate Matching Network

**Figure 8:**
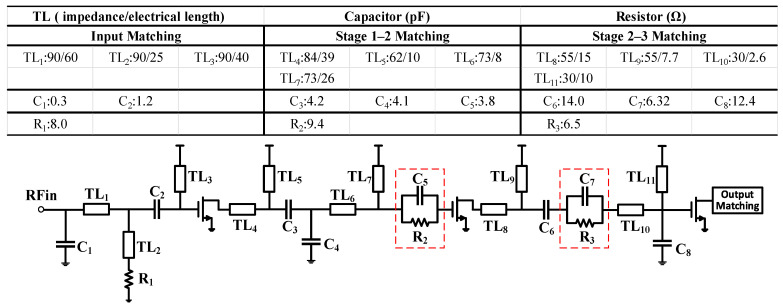
Equivalent lumped-component schematic of the input and intermediate matching networks.

The equivalent lumped-component structure of the input and interstage matching networks, along with their corresponding parameters, is shown in Figure 8. In the design of the interstage matching network, it is essential to match the output impedance of the preceding stage transistor with its optimal power impedance, ensuring sufficient power output from the preceding stage. Simultaneously, the right-side impedance must form a conjugate match with the input impedance of the subsequent stage to achieve adequate power gain.

To accomplish this, an interstage matching network composed of an RC parallel network and an LC network is employed. To enhance the flexibility of the matching network, all components contribute to impedance matching. Through a combination of calculations and tuning, the impedance is precisely conjugately matched to the input impedance of the subsequent stage transistor and aligned with the optimal power impedance of the preceding stage transistor. The RC parallel network (highlighted in the red box in Figure 8) not only broadens the bandwidth of the matching network but also allows for simultaneous adjustments to the amplifier’s stability and gain by tuning the value of the resistor. Notably, the RC parallel network between the second and third stages is placed after the LC matching network rather than directly on the gate of the transistor, as seen in conventional matching networks. This placement minimizes the resistive losses that could otherwise negatively impact the saturated output power, further enhancing the saturation performance. Additionally, *TL*_5_ and *TL*_9_ serve as drain supply lines for the first and second stages, while *TL*_7_ and *TL*_11_ act as gate supply lines for the second and third stages. These lines participate in impedance matching while also functioning as RF chokes.

### 3.3. Input Matching Network

The input matching network adopts a C-L-L-C structure, with *TL*_3_ serving as the gate supply line for the first stage and also contributing to impedance matching. To improve the bandwidth of the input impedance matching network, the resistor *R*_1_ is placed in series below *TL*_2_. By adjusting *R*_1_, a trade-off between the amplifier’s *S*_11_ and *S*_21_ can be achieved.

## 4. Measurements and Characterization

### 4.1. Test Environment Setup

**Figure 9:**
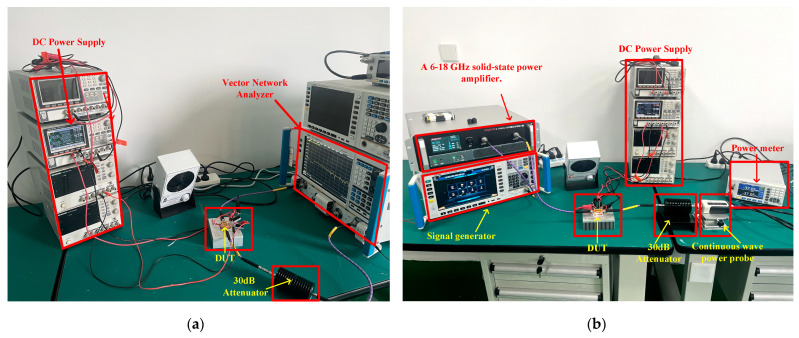
(**a**) S-parameter measurement setup; (**b**) large-signal measurement setup.

Figure 9a shows the small-signal test setup. The test is performed using a vector network analyzer, with the drain and gate voltage supplies set to 28 V and −2.2 V, respectively. A 30 dB attenuator is added to protect the instrument but is removed during the S_22_ measurement. Figure 9b illustrates the large-signal test environment. To ensure sufficient input power for measuring the saturation power of the DUT, a 6–18 GHz solid-state power amplifier is used to amplify the signal from the signal generator. This amplifier provides up to 50 W of output power and is able to measure both the forward and reflected power, meaning that the power gain can be calculated accordingly. A power meter with a continuous wave power probe measures the output power of DUT, while a 30 dB attenuator is used to protect the instrument. The DC power supply can display the DC power consumption, meaning that the PAE can be calculated accordingly.

### 4.2. Test Results Analysis

**Figure 10:**
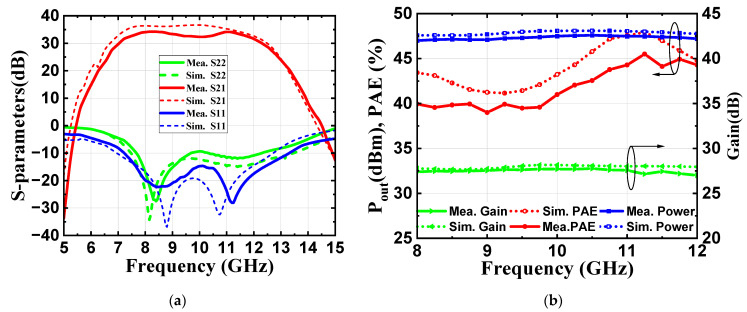
(**a**) Simulated and measured S-parameters; (**b**) simulated and measured output power, PAE, and gain versus frequency.

Figure 10a presents the measured and simulated S-parameters. As shown in Figure 10a, the input return loss (*S*_11_) remains below −14.7 dB across the operating band, the small-signal gain (*S*_21_) ranges from 32.4 to 34.2 dB, and the designed PA remains stable across all frequencies. The measured S-parameter results show a trend that is consistent with the simulation results, although there is a slight frequency shift toward higher frequencies. This minor discrepancy between the simulation and the measurements is likely due to factors such as modeling, coupling, or thermal effects, which are common in MMIC design. Therefore, during the design process, the operational bandwidth was intentionally extended and a certain margin was included to ensure that the chip maintains good performance within the target frequency range, even with potential frequency offsets and performance deterioration.

Figure 10b shows that the saturated output power ranges from 47.0 to 47.6 dBm across 8–12 GHz, with an in-band fluctuation of ±0.3 dBm. The power gain varies from 27.0 to 27.8 dB, while the PAE remains between 40.0% and 45.5% over the same frequency band. The large-signal test results generally align with the simulations, although some performance degradation is observed. This is likely due to the use of continuous wave testing, where the characteristics of the GaN power devices under high power and temperature conditions differ from those assumed in the modeling process. Therefore, such a degradation is to be expected. Despite this, the large-signal performance of the proposed amplifier remains competitive.

### 4.3. Comparison with the Existing Literature

Table 1 provides a performance comparison between the proposed X-band GaN MMIC power amplifier (PA) and other state-of-the-art designs. This comparison highlights several key parameters, such as output power, power flatness, power-added efficiency (PAE), gain, and the number of stages used in each design.

The realized PA achieves a saturated output power of 47.6 dBm across the 8–12 GHz frequency band, with an impressive in-band power flatness of ±0.3 dB. This is a notable improvement over several other designs that exhibit larger output power fluctuations, typically in the range of ±1.0 to ±1.5 dB. In terms of power-added efficiency (PAE), the proposed amplifier demonstrates an efficiency of between 40% and 45.5%, which is competitive compared to other X-band GaN MMIC amplifiers. While some designs, such as that in Ref. [17], report higher efficiencies (up to 52%), they often come at the cost of either output power or bandwidth limitations. By combining load-line theory with CCBJ, this design has precisely defined the optimal operating impedance space, which allows for a better balance between output power and efficiency. Furthermore, the well-designed RC structure enables this PA to exhibit superior gain compared to other designs.

These results validate our theoretical analysis and demonstrate the implementation of an effective design methodology for high-power, high-efficiency MMIC power amplifiers with excellent in-band *P*_out_ flatness.

## 5. Conclusions

This paper presents a novel design approach for a high-power, high-efficiency X-band GaN MMIC power amplifier, achieving significant improvements in performance and methodology. By combining load-line theory with the CCBJ impedance space, an analytical framework is developed to define the optimal impedance conditions for maximizing efficiency and minimizing power fluctuations. This approach offers a more precise and reliable path for designing high-performance power amplifiers.

The analysis of power fluctuations within the CCBJ impedance space leads to the identification of a target impedance region that ensures minimal power variation, resulting in an amplifier with excellent output power flatness. Operating across the 8–12 GHz frequency range, the amplifier delivers a saturated output power of 47.6 dBm with in-band power flatness of ±0.3 dB, outperforming similar designs in power stability.

A novel R-C interstage matching network is also introduced, enhancing the saturated output power while maintaining stability and gain. This innovation, combined with CCBJ-based impedance optimization, allows the amplifier to achieve efficiency levels of between 40% and 45.5% and a gain range of 27.0 to 27.8 dB.

In conclusion, the proposed amplifier represents a significant advancement in high-power, high-efficiency designs with superior in-band power flatness, addressing the limitations of existing amplifiers while offering a scalable approach for future X-band power amplifier development.

## Figures and Tables

**Table 1:** Performance comparison with X-band GaN MMIC PAs.

Ref.	Process	Freq.	P_out_	P_out_ Flatness	PAE	Gain	Stages	Area	Class
	(µm)	(GHz)	(dBm)	(dBm)	(%)	(dB)	(\)	(mm^2^)	(\)
[1]	0.25	9.8–10.4	42	±1.1	38	18	2	18	AB
[2]	0.25	8.5–10.2	43	±1.2	43	25	3	7.7	F^−1^
[4]	0.14	9.6–11.0	40	±1.5	61	10	1	\	F
[14]	0.25	8–12	43	±0.5	38	23	3	7.5	AB
[15]	0.25	8–12	49	±0.55	45	22.7	3	13.3	AB
[16]	0.15	10–10.5	42	±0.75	50	20	2	9.2	HT_3_
[17]	0.15	8–12.5	41	±0.60	52	20	2	\	AB
[18]	0.15	9.5–10.5	47	±1.0	55	10	1	12.2	AB
This work	0.25	8–12	47.6	±0.3	45.5	27.8	3	17	J

## Data Availability

The data presented in this work are available within the article.
